# Older adults’ transportation walking: a cross-sectional study on the cumulative influence of physical environmental factors

**DOI:** 10.1186/1476-072X-12-37

**Published:** 2013-08-14

**Authors:** Jelle Van Cauwenberg, Peter Clarys, Ilse De Bourdeaudhuij, Veerle Van Holle, Dominique Verté, Nico De Witte, Liesbeth De Donder, Tine Buffel, Sarah Dury, Benedicte Deforche

**Affiliations:** 1 Department of Human Biometry and Biomechanics, Faculty of Physical Education and Physical Therapy, Vrije Universiteit Brussel, Pleinlaan 2, B-1050 Brussels, Belgium; 2 Department of Movement and Sport Sciences, Faculty of Medicine and Health Sciences, Ghent University, Watersportlaan 2, B-9000 Ghent, Belgium; 3 Fund for Scientific Research Flanders Belgium (FWO), Egmontstraat 5, B-1000 Brussels, Belgium; 4 Department of Educational Sciences, Faculty of Psychology and Educational Sciences, Vrije Universiteit Brussel, Pleinlaan 2, B-1050 Brussels, Belgium; 5 University College Ghent, Keramiekstraat 78-80, 9000 Gent, Belgium; 6 School of Social Science, The University of Manchester, Oxford Road, Manchester M13 9PL United Kingdom

**Keywords:** Ecological model, Environment, Physical activity, Walking, Older adults

## Abstract

**Background:**

The physical environment may play a crucial role in promoting older adults’ walking for transportation. However, previous studies on relationships between the physical environment and older adults’ physical activity behaviors have reported inconsistent findings. A possible explanation for these inconsistencies is the focus upon studying environmental factors separately rather than simultaneously. The current study aimed to investigate the cumulative influence of perceived favorable environmental factors on older adults’ walking for transportation. Additionally, the moderating effect of perceived distance to destinations on this relationship was studied.

**Methods:**

The sample was comprised of 50,685 non-institutionalized older adults residing in Flanders (Belgium). Cross-sectional data on demographics, environmental perceptions and frequency of walking for transportation were collected by self-administered questionnaires in the period 2004-2010. Perceived distance to destinations was categorized into short, medium, and large distance to destinations. An environmental index (=a sum of favorable environmental factors, ranging from 0 to 7) was constructed to investigate the cumulative influence of favorable environmental factors. Multilevel logistic regression analyses were applied to predict probabilities of daily walking for transportation.

**Results:**

For short distance to destinations, probability of daily walking for transportation was significantly higher when seven compared to three, four or five favorable environmental factors were present. For medium distance to destinations, probabilities significantly increased for an increase from zero to four favorable environmental factors. For large distance to destinations, no relationship between the environmental index and walking for transportation was observed.

**Conclusions:**

Our findings suggest that the presence of multiple favorable environmental factors can motivate older adults to walk medium distances to facilities. Future research should focus upon the relationship between older adults’ physical activity and multiple environmental factors simultaneously instead of separately.

## Background

Physical inactivity is a major threat to the physical, mental and social health of the growing population of older adults (≥ 65 years) [1-4]. Walking for transportation is a healthy and acceptable activity that is easy to integrate in an older adult’s daily routine (e.g. walking to a shop) [5]. Ecological models state that, in order to promote walking for transportation, interventions should target individual as well as physical, social and policy environmental factors [6-8]. Physical environmental factors are especially relevant for older adults as age-related functional limitations can cause problems in overcoming physical barriers (e.g. distance, obstacles, etc.) [9-11].

A systematic review retrieved six studies on the relationship between the physical environment and older adults’ transportation walking. These studies showed inconsistent results concerning the relationships between walking for transportation and access to public transport, presence and quality of walking facilities, traffic- and crime-related safety and aesthetics [12]. A possible explanation for these inconsistencies is the focus on studying relationships between walking for transportation and multiple environmental factors separately instead of combined. It seems plausible that older adults’ walking for transportation is not influenced by merely the presence of one favorable environmental factor (e.g. an even sidewalk). Rather, it may be the cumulative influence of multiple favorable environmental factors (e.g. an even sidewalk, safe crossings, safety from crime, etc.) that affect older adults’ choice to walk or not in a certain environment. For example in adults, Sallis and colleagues [13] reported that the presence of at least four favorable environmental factors was required to find a significant relationship with overall physical activity levels.

In several recent studies the objective and perceived presence of nearby destinations (e.g. shops, services, etc.) appeared to be a strong positive predictor of older adults’ walking for transportation [14-17]. For example, Frank and colleagues [14] reported residents of high-walkable neighborhoods (many destinations within walking distance) to be twice as likely to walk for transportation compared to residents of low-walkable neighborhoods (few destinations within walking distance). However, distances to destinations are difficult to modify in existing neighborhoods. Therefore, it is crucial to know whether the presence of one or more favorable and modifiable environmental factors can stimulate older adults to walk even when destinations are located further away. According to Alfonzo’s “Hierarchy of walking needs” access/distance to destinations is the most basic need that has to be fulfilled before an older adult will walk for transportation [18]. Consequently, other environmental factors can be expected to be unrelated to older adults’ walking for transportation in areas where access to destinations is low.

To our knowledge, no previous study has investigated the cumulative influence of environmental factors on older adults’ PA behaviors, neither has a study focused upon the possible moderating effect of distance to destinations. Therefore, the current study aimed to investigate the cumulative influence of the perceived presence of favorable environmental factors on older adults’ walking for transportation. It was hypothesized that there exists a cumulative influence of perceived favorable environmental factors on older adults’ walking for transportation, such that when more favorable environmental factors were present, older adults would walk for transportation more often. Additionally, the moderating effect of perceived distance to destinations on this relationship was studied. We hypothesized that the cumulative influence of the perceived presence of favorable environmental factors on older adults’ walking for transportation would disappear when perceived distance to destinations was large.

## Methods

### Sampling and data collection

Data were derived from the Belgian Ageing Studies (BAS). Detailed information on data collection has been reported previously [17,19-21]. Briefly, non-institutionalized persons aged 60 years or older were randomly sampled, stratified for age and gender, from the population registers of the participating municipalities. Municipalities were not selected randomly, they could freely decide to participate in the study. Consequently, the final sample was not representative at a national level, but every sample was representative for the specific municipality. The participating 142 municipalities did not differ in average yearly income/inhabitant (16452 euros) from the average of all 308 Flemish municipalities (16323 euros), but were more densely populated (572 vs. 457 inhabitants/km^2^). Peer research methodology was used to collect data. This means that older adults were not only involved in the study as participants but that they were also actively involved in the research process, i.e. they collected the data. Older volunteers were recruited within their municipalities and attended several training sessions. These older volunteers delivered and collected self-administered questionnaires in their peer group. If requested, questionnaires were available in six other languages than Dutch: French, Spanish, Arabic, Russian, Turkish, and Italian. Peer research minimizes social desirability and results in more complete questionnaires and higher response rates [22]. In the present study, response rates ranged from 65 to 85%, depending on municipality. 67,563 community-dwelling persons within 142 of the 308 municipalities in the region of Flanders (Belgium) agreed to participate. Data collection was performed between 2004 and 2010.

Consistent with the definition of older adults in international PA guidelines [2], only respondents aged ≥ 65 years were included, resulting in a final sample of 50,685 older adults within 142 municipalities. The study was approved by the ethical committee of the hospital of the Vrije Universiteit Brussel (B.U.N. 143201111521, 17/07/2011).

### Measures

#### Demographic variables

The following demographic covariates were assessed: age, gender, marital status, educational level and monthly income. Number of functional limitations was also assessed since they have been shown to be related to older adults’ PA behaviors [23,24]. Number of functional limitations were measured by asking whether or not participants were limited in performing the following seven activities of daily living: vigorous activities, moderate activities, climb several flights, bend/kneel, walk one block, bath/dress, and household chores. These seven items are similar to the “physical functioning” subscale of the validated SF-36 [25,26]. Activities in which participants reported to be limited were summed to obtain “number of functional limitations”.

#### Walking for transportation

Walking for transportation was assessed by the following question: “How often do you walk for transportation?” Respondents answered on a 5-point scale ranging from “never” to “almost daily”. Consistent with the international guidelines of being moderately-to-vigorously physically active on at least 5 days/week [2] scores were dichotomized into “almost daily walking for transportation” versus “less than almost daily walking for transportation”. For reasons of convenience and readability the dependent variable was entitled “daily walking for transportation”.

#### Environmental variables

Municipalities’ residential densities in 2008 were obtained from the Study Service of the Flemish Government [27]. Municipalities were categorized as rural (residential density ≤ 150 inhabitants/km^2^), semi-rural (150-300 inhabitants/km^2^), semi-urban (300-600 inhabitants/km^2^) and urban (> 600 inhabitants/km^2^) [28]. As rural areas are scarce in Flanders, rural and semi-rural areas were collapsed into one category “rural areas”.

To investigate the cumulative influence of perceived favorable environmental factors (measured at the individual level), an environmental index was constructed. This environmental index included the following seven environmental factors: (1) absence of high curbs, presence of (2) different shops and services, (3) benches, (4) crossings, (5) bus stops, and (6) street lighting, and (7) safety from crime. These environmental factors were included in the index because they were positively related or unrelated to walking for transportation in a previous analysis on the same dataset [17]. The included perceived environmental factors variables were assessed as follows. (1) Absence of high curbs was assessed through: “How applicable is the following statement to your neighborhood? There are high curbs present in and around my house”. A 5 point-scale ranging from “completely not applicable” to “completely applicable” was provided. (2) Presence of 13 different kinds of shops and services (e.g. grocery store, pharmacy, post office…), (3) benches, (4) crossings, (5) bus stops and (6) street lighting were measured by a single-item question: “Are the following facilities sufficiently present in your neighborhood?” Answer categories were “yes” or “no”. Responses on answers concerning the presence of 13 different kinds of shops and services were summed to create the variable “number of shops”. (7) Safety from crime was measured by the “Elderly Feelings of Unsafety” (EFU) scale [19]. Ordinal and quantitative perceived environmental factors were dichotomized around their median. The seven included factors were dummy coded such that value “1” represented the anticipated favorable aspect of the environmental factor to be present (e.g. no curbs present, benches present, safe from crime). The environmental index was constructed by summing the dummy coded scores on the seven environmental variables. Hence, the environmental index ranged from zero (no favorable environmental factors were present) to seven (all favorable environmental factors were present).

Perceived distance to destinations was assessed through: “How applicable is the following statement to your neighborhood? Facilities (e.g. shop, bank, etc.) are located within short distances from my home.” A 5 point-scale ranging from “completely inapplicable” to “completely applicable” was provided. Scores 1-2, 3-4 and 5 were recoded into “large”, “medium” and “short distances to facilities”, respectively.

### Analysis

Taking account of the hierarchical data structure (participants nested within municipalities), multilevel analyses (i.e. mixed models) were applied using the MLwiN 2.24 software. All variables were measured at the individual level, except for residential density (area of residence) which was measured at the municipality-level. Logistic regression was used to predict probabilities of daily walking for transportation. Model parameter estimates were obtained via Markov Chain Monte Carlo (MCMC) procedures applying an orthogonal parameterization. First, the separate relationships between the seven environmental variables and walking for transportation were analyzed and odds ratios with corresponding Bayesian confidence intervals were reported. Secondly, a model was built including the environmental index, distance to destinations and their interaction terms. During data exploration, a curvilinear relationship was observed between the environmental index and probabilities of daily walking for transportation. Therefore, a quadratic term was added to the model (indicated as environmental index^2^). The effects of the index were interpreted by using predicted population-averaged probabilities [29-31]. To mutually compare the 24 predicted probabilities (3 distance categories × 8 possible scores on the environmental index), 95% confidence intervals are presented in table and graph format. All analyses were adjusted for gender, age, marital status, number of functional limitations, education, monthly income and area of residence (rural, semi-urban or urban). MLwiN uses listwise deletion, consequently, participants with missing data on any variable in the model were not included in the analysis. This resulted in the inclusion of 27,693 participants in the final model. Participants included were less likely to be female (60.0 vs. 51.7% females), slightly younger (73.7 vs. 74.8 years), better educated (12.6 vs. 6.8% had tertiary education), and walked more (38.4 vs. 32.4% walked for transportation daily) compared to those that were not included. Significance level was set at 0.05.

## Results

### Descriptive statistics

Descriptive statistics are presented in Table 1. Participants reported a mean of 4.7 (± 1.6) favorable environmental factors to be present in their neighborhood. 54.3% of the participants perceived facilities to be within short distances from their home. 34.9% of the participants reported to walk for transportation daily.

**Table 1:** Descriptive statistics

**Variables**	**Descriptives**
Age (M ± SD)	74.2 ± 6.4
Gender (% female)	55.5
Marital status (%)	
Married / cohabiting	68.0
Divorced / never married	6.2
Widowed	25.8
Educational level (%)	
Primary	46.1
Lower secondary	27.7
Higher secondary	16.1
Tertiary	10.0
Monthly Income (%)	
500 - 999 euro	26.4
1000 - 1499 euro	38.2
1500 - 1999 euro	19.9
≥ 2000 euro	15.6
Number of functional limitations^1^ (M ± SD)	2.5 ± 2.6
Area of residence (%)	
Rural	32.7
Semi-urban	34.8
Urban	32.5
Environmental index^2^ (M ± SD)	4.7 ± 1.6
Distance to services^3^ (%)	
Large	22.3
Medium	23.4
Short	54.3
Daily walking for transportation (%)	34.9

*M* mean, *SD* Standard deviation.

^1^Number of activities of daily living in which participants reported to be limited (range: 0 - 7).

^2^Number of favorable environmental factors participants reported to be present in their neighborhood (range: 0 - 7).

^3^“Facilities (e.g. shop, bank, etc.) are located within short distances from my home”. Large= completely inapplicable/inapplicable; medium= neutral/applicable; short= completely applicable.

### Separate relationships between the seven environmental variables and walking for transportation

The following four environmental variables were significantly positively related to walking for transportation: presence of bus stops, street lighting, number of shops and safety from crime. No significant relationships were observed for absence of high curbs and presence of benches and crossings (see Table 2).

**Table 2:** Relationships between walking for transportation and the environmental correlates separately

**Environmental factors**^**a**^	**Odds ratio (95% C.I.)**
Absence of high curbs	1.00 (0.94, 1.06)
Number of shops	1.20 (1.15, 1.26)*
Presence of benches	0.98 (0.93, 1.03)
Presence of crossings	1.01 (0.95, 1.07)
Presence of bus stops	1.29 (1.22, 1.37)*
Presence of street lighting	1.12 (1.04, 1.19)*
Safety from crime	1.08 (1.03, 1.13)*

*C.I.* (Bayesian) Confidence Interval.

All analyses were adjusted for gender, age, marital status, number of functional limitations, education, monthly income, and area of residence.

^a^All environmental factors were dichotomous and dummy coded with “1” being the anticipated favorable aspect of the factor.

*p< 0.05.

### Relationships between the environmental index and walking for transportation and the moderating effect of distance to destinations

**Figure 1:**
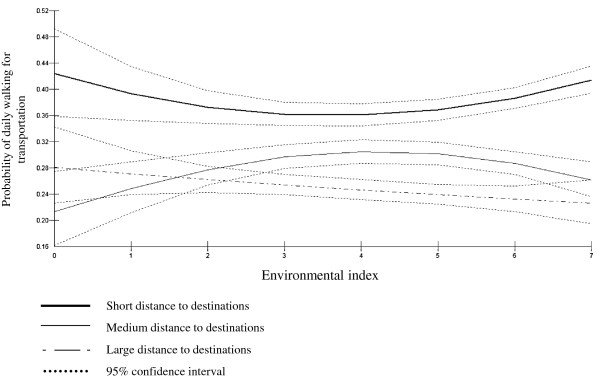
Predicted probabilities of daily walking for transportation.

Table 3 presents the results of the full model. Predicted probabilities and 95% confidence intervals of daily walking for transportation by environmental index and distance to destinations are presented in Table 4 and displayed graphically in Figure 1. Probabilities of daily walking for transportation were significantly higher for participants living within short distances (prob. ranging from 0.36 - 0.42) compared to medium (prob. ranging from 0.22 - 0.31) and large distances to facilities (prob. ranging from 0.23 - 0.28). A significant interaction effect between the environmental index and distance to destinations was observed. Therefore, the results are described separately for each category of perceived distance to destinations.

**Table 3:** Model explaining daily walking for transportation including the environmental index, distance to destinations and their interaction terms

	**β**	**S.E.**	**95% C.I.**
Constant	−0.632	0.157	(-0.941, -0.325)*
Age (GM)	0.004	0.002	(-0.001, 0.009)
Gender (ref. = female)	0.222	0.027	(0.169, 0.275)*
Marital status (ref. = widowed)			
Married / Cohabiting	0.306	0.059	(0.190, 0.421)*
Living alone / divorced	−0.103	0.035	(-0.173, -0.034)*
Functional limitations	−0.151	0.006	(-0.162, -0.139)*
Educational level (ref.= primary education)			
Lower secondary education	0.067	0.032	(0.003, 0.130)*
Higher secondary education	0.055	0.038	(-0.021, 0.130)
Tertiary education	0.085	0.047	(-0.006, 0.177)
Income (ref. = 500 - 999 euro)			
1000 - 1499 euro	0.115	0.036	(0.045, 0.185)*
1500 - 1999 euro	0.128	0.043	(0.045, 0.211)*
≥ 2000 euro	0.012	0.048	(-0.082, 0.108)
Area of residence (ref. = rural)			
Semi-urban	0.047	0.060	(-0.072, 0.164)
Urban	0.306	0.062	(0.186, 0.427)*
Environmental index			
Environmental index	−0.046	0.078	(-0.199, 0.107)
Environmental index^2^	0.001	0.010	(-0.019, 0.020)
Distance to destinations (ref.= large distance)			
Medium distance	−0.376	0.225	(-0.822, 0.065)
Short distance	0.626	0.202	(0.230, 1.021)*
Interaction terms			
Environmental index * medium distance	0.282	0.113	(0.062, 0.505)*
Environmental index^2^ * medium distance	−0.029	0.013	(-0.055, -0.002)*
Environmental index * short distance	−0.103	0.099	(-0.297, 0.093)
Environmental index^2^ * short distance	0.020	0.012	(-0.003, 0.043)
**Variances**	**Var/covar**	**S.E.**	**95% C.I.**
Level: municipality			
Constant/constant	0.062	0.013	(0.040, 0.091)*
Short distance/constant	−0.013	0.011	(-0.038, 0.007)
Short distance/short distance	0.039	0.015	(0.015, 0.075)*
−2*loglikelihood:			
DIC:	34838.265		
pD:	157.533		

*S.E.* Standard Error, *C.I*. (Bayesian) Confidence Interval, *GM* centered around its grand mean, *DIC* Deviance Information Criterium; pD= estimated degrees of freedom consumed in fitting the model. * p< 0.05.

**Table 4:** Predicted probabilities of daily walking for transportation

**Environmental index**	**Short distance**	**Medium distance**	**Large distance**
	**Prob. (95% C.I.)**	**Prob. (95% C.I.)**	**Prob. (95% C.I.)**
0	0.42 (0.36, 0.49)^a,b^	0.22 (0.16, 0.28)^c,e^	0.28 (0.23, 0.34)^c,d,f^
1	0.39 (0.35, 0.43)^a,b^	0.25 (0.22, 0.29)^c,d,e^	0.27 (0.24, 0.31)^c,d,f^
2	0.37 (0.34, 0.39)^a,b^	0.28 (0.26, 0.31)^c,d,e^	0.27 (0.25, 0.29)^c,d,f^
3	0.36 (0.34, 0.38)^a^	0.30 (0.28, 0.32)^c,d^	0.26 (0.24, 0.27)^c,f^
4	0.36 (0.34, 0.38)^a^	0.31 (0.29, 0.33)^d^	0.25 (0.24, 0.27)^c,f^
5	0.37 (0.35, 0.38)^a^	0.30 (0.29, 0.32)^d^	0.24 (0.23, 0.26)^e,f^
6	0.38 (0.37, 0.40)^a,b^	0.29 (0.27, 0.31)^c,d^	0.24 (0.22, 0.26)^e,f^
7	0.41 (0.39, 0.43)^b^	0.26 (0.24, 0.29)^c,d,e^	0.23 (0.20, 0.26)^e,f^

*Prob.* Probability, *C.I.* Confidence Interval.

^a,b,c,d,e,f^Across columns and rows, probabilities with the same indices do not differ significantly (α= 0.05).

### Short distance to destinations

Within short distances to facilities, the largest probabilities were observed for the presence of 0 and 7 favorable environmental factors. However, for a low number of favorable environmental factors confidence intervals were wide (e.g. for 0 favorable environmental factors 95% C.I.: 0.36, 0.49). Probabilities of daily walking for transportation started to increase when at least five favorable environmental factors were present such that the probability of daily walking for transportation was significantly higher for seven (prob.= 0.41; 95% C.I.: 0.39,0.43) compared to three (prob.= 0.36; 95% C.I.: 0.34, 0.38), four (prob.= 0.36; 95% C.I.: 0.34, 0.38) or five (prob.= 0.37; 95% C.I.: 0.35, 0.38) favorable environmental factors.

### Medium distance to destinations

For medium distance to destinations, probabilities significantly increased from 0.22 (95% C.I.: 0.16, 0.28) for zero environmental factors to 0.31 (95% C.I.: 0.29, 0.33) for four and 0.30 (95% C.I.: 0.29, 0.32) for five favorable environmental factors. Probabilities of daily walking for transportation tended to decrease for six (prob.= 0.29; 95% C.I.: 0.27, 0.31) and seven (prob.= 0.26; 95% C.I.: 0.24, 0.29) favorable environmental factors, but this decrease was not significant.

If a maximum of two favorable environmental factors were present no significant differences in probabilities of daily walking for transportation were observed between participants who perceived distance to destinations to be medium (prob.= 0.28; 95% C.I.: 0.26, 0.31) versus large (prob.= 0.27; 95% C.I.: 0.25, 0.29). If more than two favorable environmental factors were present, probabilities were significantly higher for perceived medium distance compared to large distance to destinations. However, when seven favorable environmental factors were present no significant difference in probabilities of daily walking for transportation between medium (prob.= 0.26; 95% C.I.: 0.24, 0.29) and large distance to destinations (prob.= 0.23; 95% C.I.: 0.20, 0.26) was observed.

### Large distance to destinations

For large distance to destinations, a non-significant decrease from 0.28 (95% C.I.: 0.23, 0.34) to 0.23 (95% C.I.: 0.20, 0.26) in the probability of daily walking for transportation was observed for an increase from zero to seven favorable environmental factors.

It should be noted that for medium and large distances the uncertainty for lower and higher scores on the index is larger because of widening confidence intervals.

## Discussion

To our knowledge this is the first study to investigate the cumulative influence of favorable environmental factors on older adults’ walking for transportation by categories of perceived distance to destinations. Our hypothesis that there is a cumulative effect of favorable environmental factors on older adults’ walking for transportation was partially confirmed by our findings. A cumulative relationship was observed when participants lived within short, but especially within medium distance to destinations. However, in line with our second hypothesis, a cumulative relationship was absent when participants lived within large distance to destinations.

Independent of the presence of other favorable environmental factors, participants living within short distance to destinations were more likely to walk daily for transportation. This is consistent with several recent studies reporting a positive relationship between older adults’ walking for transportation and the perceived and objective presence of nearby facilities [14-16]. Hence, recent changes in local communities’ structures, particularly in terms of the closure of facilities and services (e.g. post offices, bakeries) [32], might negatively affect older adults’ walking for transportation. An increase in probabilities of daily walking for transportation was only observed when at least seven favorable environmental factors were present. It seems that a short distance to destinations is an important facilitator of walking for transportation and that an accumulation of multiple favorable environmental factors (seven in the current study) are needed to further stimulate walking for transportation.

A clear cumulative effect of favorable environmental factors was observed among participants who perceived facilities to be located within medium distances from their homes. Our findings showed that at least four favorable environmental factors needed to be present to find a significant influence on older adults’ walking for transportation. The presence of additional favorable environmental factors did not further increase probabilities of daily walking for transportation. This is similar to the results reported by Sallis and colleagues [13] who found that at least four favorable environmental factors needed to be present to find a significant relationship with total PA levels in adults. Two very recent studies also reported similar (curvilinear) dose-response relationships between environmental indices and adults’ total sitting time, motorized transport [33] and walking and cycling for transportation [34]. Our findings suggest that a favorable walking environment can motivate older adults to walk moderate distances to facilities. However, they also suggest that the modification of single environmental characteristics are unlikely to result in increasing levels of older adults' walking for transportation unless other favorable environmental factors are already in place. This implies that environmental programs to promote walking for transportation should apply different strategies depending on the target area. How many and which environmental factors should be modified will depend on the environmental features that are already present. Based upon our findings it is not possible to state if certain combinations of environmental factors are more or less effective to promote walking for transportation compared to other combinations. Future research is needed to investigate this issue.

No relationship between the presence of favorable environmental factors and daily walking for transportation was observed if participants perceived distance to destinations to be large. Apparently, a large distance to destinations forms such a strong deterrent to daily walking for transportation that it cannot be overcome by the presence of multiple favorable environmental factors. Similarly, other deterrents (e.g. low perceived safety from crime) that cannot be overcome by the presence of one or more favorable environmental might exist. Such interaction effects should be investigated in future research. Promoting walking for transportation by means of environmental modifications in areas with restricted access to destinations might involve supporting maintenance of local shops and services and attracting new shops and services (e.g. through tax incentives). Furthermore, the possibility of changing older adults’ perception of a “walkable” distance and its effects on walking for transportation should be explored.

Several strengths and weaknesses of this study should be considered. A first strength is the investigation of the cumulative influence of multiple favorable environmental factors on older adults’ walking for transportation rather than the influence of each environmental factor separately. Studying the moderating effect of distance to destinations on this relationship is another innovation in this research area. Secondly, these analyses were carried out on a large sample of older adults. However, despite the large sample size, caution is needed while interpreting the results. Few participants reported a low and high number of favorable environmental factors which resulted in a widening of the confidence intervals for these values. Future research including very unfavorable and favorable walking environments is needed to confirm current findings. Furthermore, residential densities in the participating municipalities were higher than in the average Flemish municipality. Since Flanders is already a very densely populated region, research in other (less dense) countries is needed to confirm current findings. This emphasizes the need for (international) studies which cover a wide variety of environmental contexts. A second limitation is the absence of information on the psychometrics of the measure of walking for transportation. Furthermore, this measure captured frequency but not duration or intensity of walking for transportation. Thirdly, this study relied on subjective rather than objective environmental measures on a limited set of environmental factors. For example, this study did not include a measure of aesthetic features of the environment. Finally, inferring causal conclusions is not possible because of the cross-sectional study design.

In conclusion, there appears to be a cumulative influence of physical environmental factors on older adults’ walking for transportation. However, this relationship was moderated by distance to destinations. These cumulative effects of and interaction effects between environmental factors offer a possible explanation for the inconsistencies between previous studies [12]. Our findings highlight the need for future research to study the relationship between older adults’ PA and multiple environmental factors simultaneously instead of separately. Further research should reveal which combinations of environmental factors need to be present to optimally stimulate older adults’ walking for transportation in areas with different access to facilities. If the current findings are confirmed, interventions should not only target multiple levels, such as the person within his social, physical and policy environment [8], but also multiple factors within the physical environment.

## Competing interest

The authors declare that they have no competing interests.

## Authors’ contributions

DV, NDW, LDD, TB, and SD developed the design of the Belgian Aging Studies and were responsible for data collection. JVC, PC, IDB, VVH, DV, LDD, and BD designed the protocol for this (sub)study. JVC performed the statistical analyses and drafted the manuscript. JVC, PC, IDB, VVH, DV, LDD, and BD critically revised and helped to draft the manuscript. All authors read and approved the final manuscript.
